# Supplementary material for: Stratification of Gut Microbiota Profiling Based on Autism Neuropsychological Assessments
Source: Microorganisms. 2024 Oct 9;12(10):2041. doi: 10.3390/microorganisms12102041 (PMC11510388; doi:10.3390/microorganisms12102041)
Supplement: Supplementary file 1 [file microorganisms-12-02041-s001.zip › Figure S1.pdf]

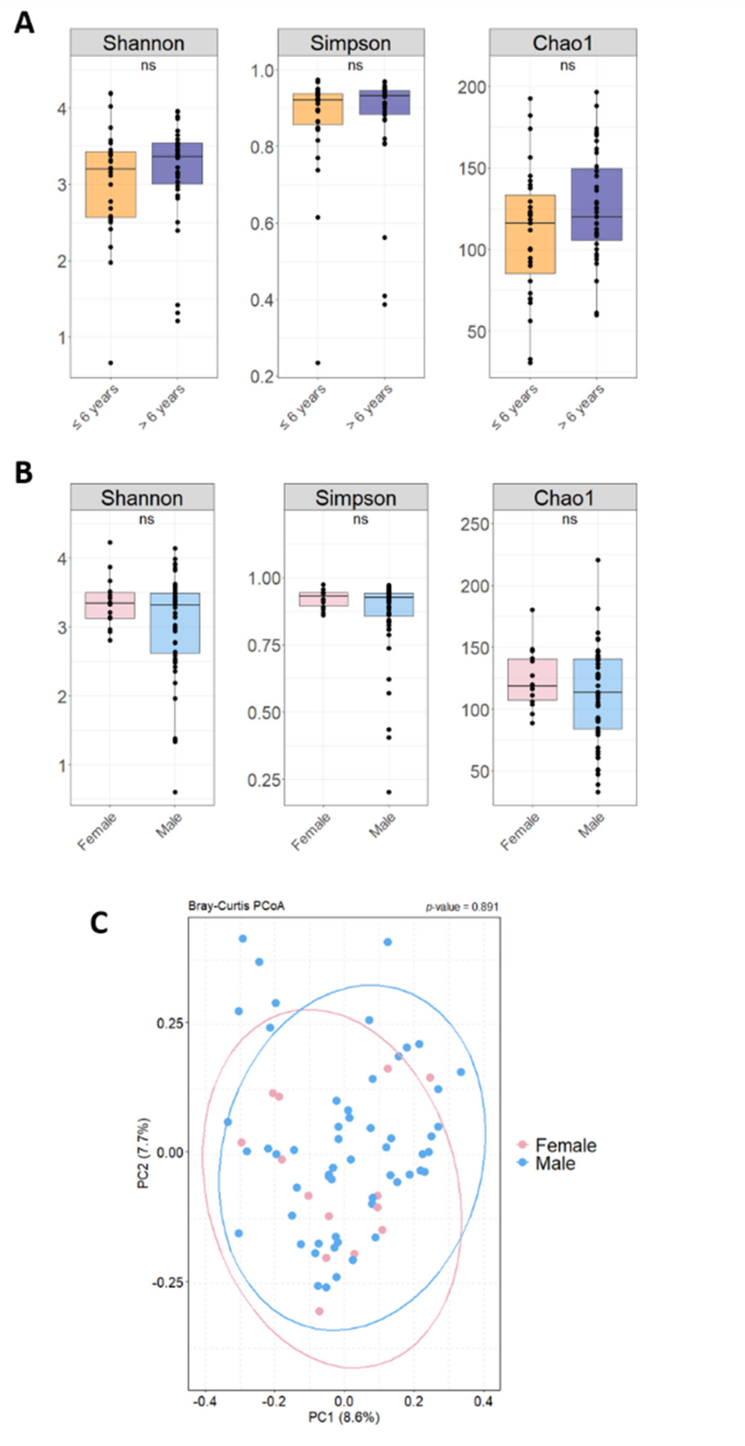

Supplementary Figure 1. Ecological analyses of ASD patients grouped by potential confounding factor age and gender. Alpha diversity based on Shannon, Simpson and Chao1 indices of ASD patients grouped by age **(A)** and gender **(B)**. The pairwise-comparisons were obtained by a post hoc Mann–Whitney test (ns: p-value > 0.05).

Beta-diversity analysis based on Bray–Curtis dissimilarity algorithm calculated for ASD patients stratified by gender **(C)**. PERMANOVA p-value is reported.
