# Supplementary material for: Stratification of Gut Microbiota Profiling Based on Autism Neuropsychological Assessments
Source: Microorganisms. 2024 Oct 9;12(10):2041. doi: 10.3390/microorganisms12102041 (PMC11510388; doi:10.3390/microorganisms12102041)
Supplement: Supplementary file 1 [file microorganisms-12-02041-s001.zip › Figure S2.pdf]

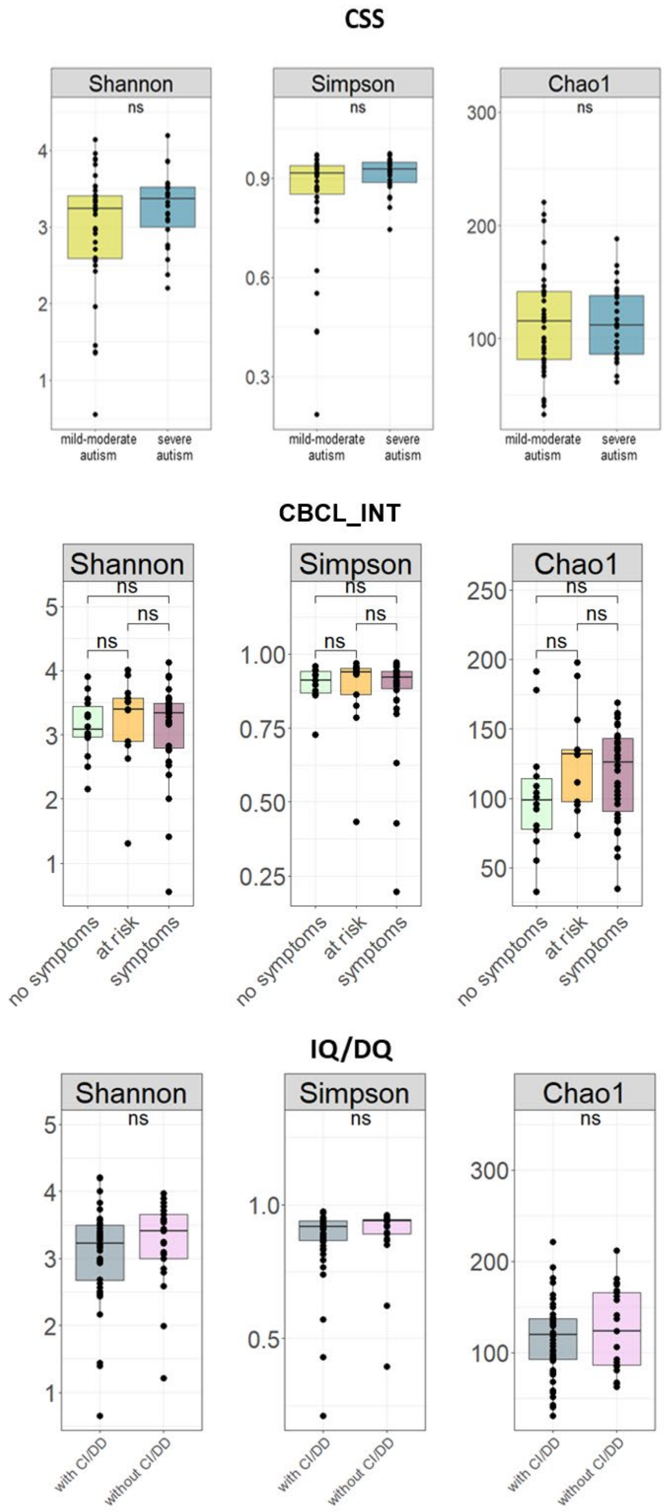

Supplementary Figure 2. Alpha diversity of ASD patients grouped by CSS, CBCL_INT and IQ/DQ features. Alpha-diversity was based on Shannon, Simpson and Chao1 indices. Comparisons amongst three sub-groups (no symptoms; at risk; and symptoms) were obtained by Kruskal-Wallis test, while the sub-group pairwise-comparisons through a post hoc Mann–Whitney test (ns: p-value > 0.05).
