# Supplementary material for: Stratification of Gut Microbiota Profiling Based on Autism Neuropsychological Assessments
Source: Microorganisms. 2024 Oct 9;12(10):2041. doi: 10.3390/microorganisms12102041 (PMC11510388; doi:10.3390/microorganisms12102041)
Supplement: Supplementary file 1 [file microorganisms-12-02041-s001.zip › Figure S3.pdf]

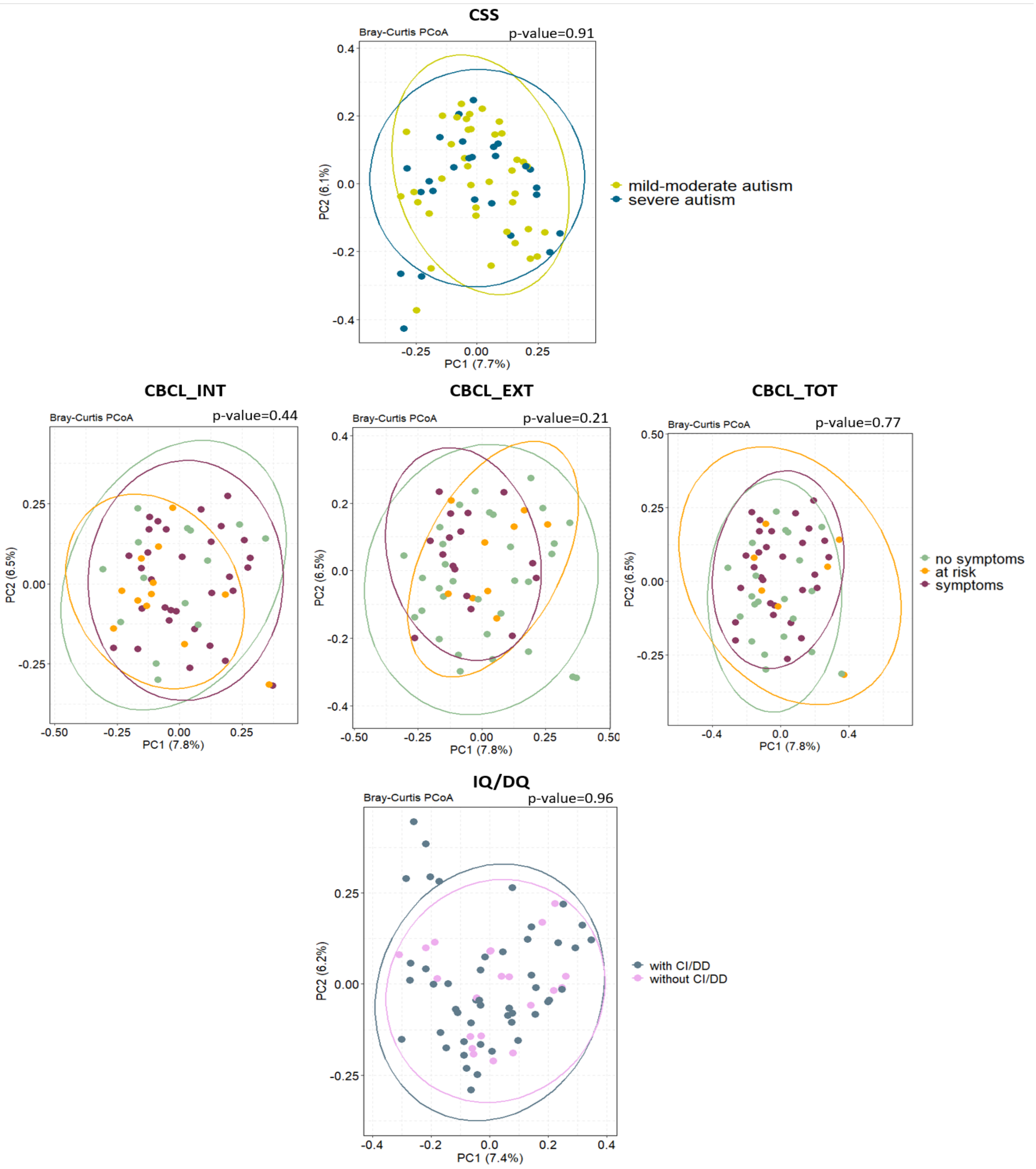

Supplementary Figure 3. Beta diversity of ASD patients grouped by clinical features. Principal Coordinate Analysis (PCoA) of beta-diversity was computed by patients grouping based on CSS, CBCL_INT, CBCL_EXT, CBCL_TOT and IQ/DQ features, performed by Bray-Curtis algorithm.

In figure, the resulting p-values for PERMANOVA analyses.
