# Supplementary material for: Stratification of Gut Microbiota Profiling Based on Autism Neuropsychological Assessments
Source: Microorganisms. 2024 Oct 9;12(10):2041. doi: 10.3390/microorganisms12102041 (PMC11510388; doi:10.3390/microorganisms12102041)
Supplement: Supplementary file 1 [file microorganisms-12-02041-s001.zip › Figure S4.pdf]

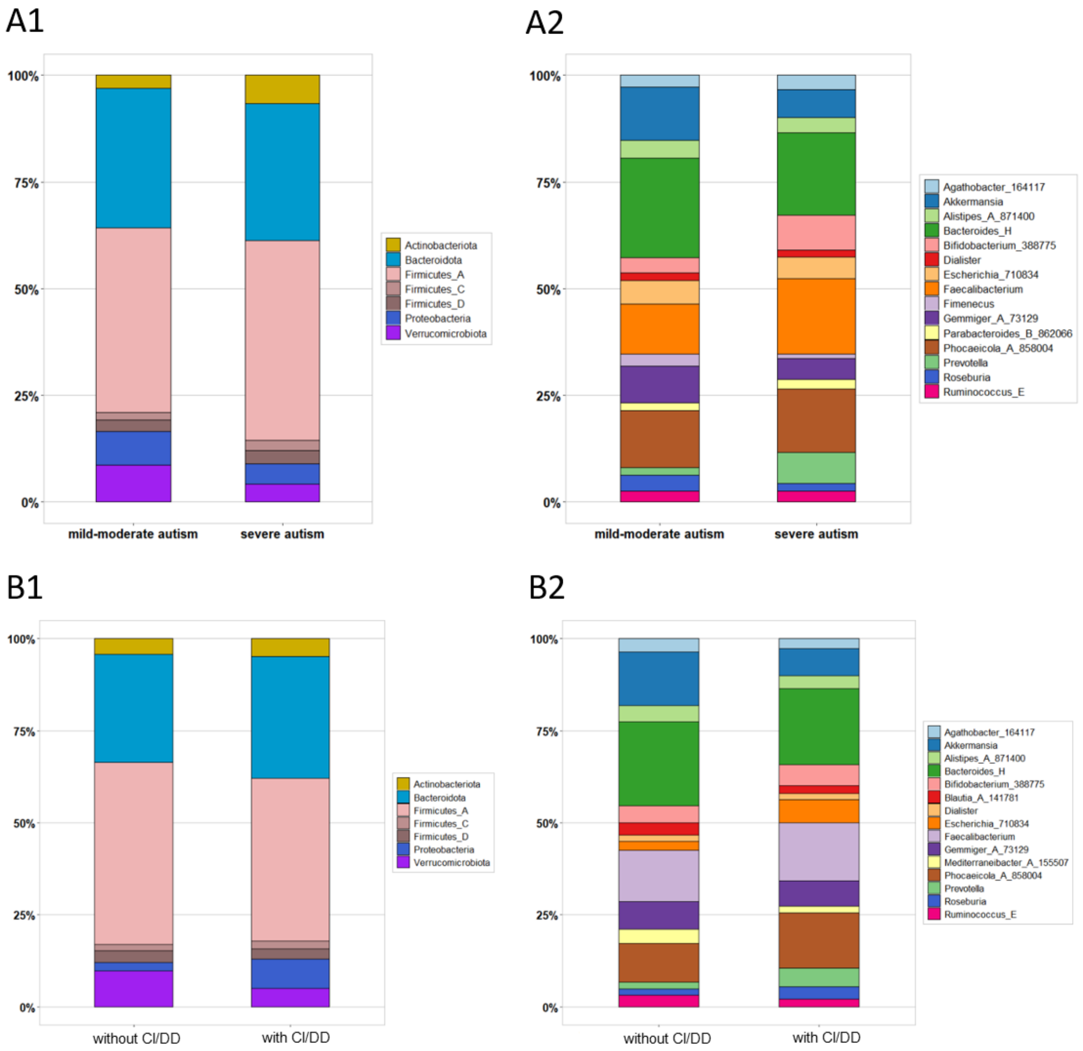

Supplementary Figure 4. Gut microbiota profiles of ASD patients stratified by CSS (A) and IQ/DQ (B). Abundance of phyla (A1 and B1 panels) and top 15 genera (A2 and B2 panels) were expressed as relative percentage for ASD patients stratified by CSS and IQ/DQ neuropsychological features.
