# Supplementary material for: Stratification of Gut Microbiota Profiling Based on Autism Neuropsychological Assessments
Source: Microorganisms. 2024 Oct 9;12(10):2041. doi: 10.3390/microorganisms12102041 (PMC11510388; doi:10.3390/microorganisms12102041)
Supplement: Supplementary file 1 [file microorganisms-12-02041-s001.zip › Figure S5.pdf]

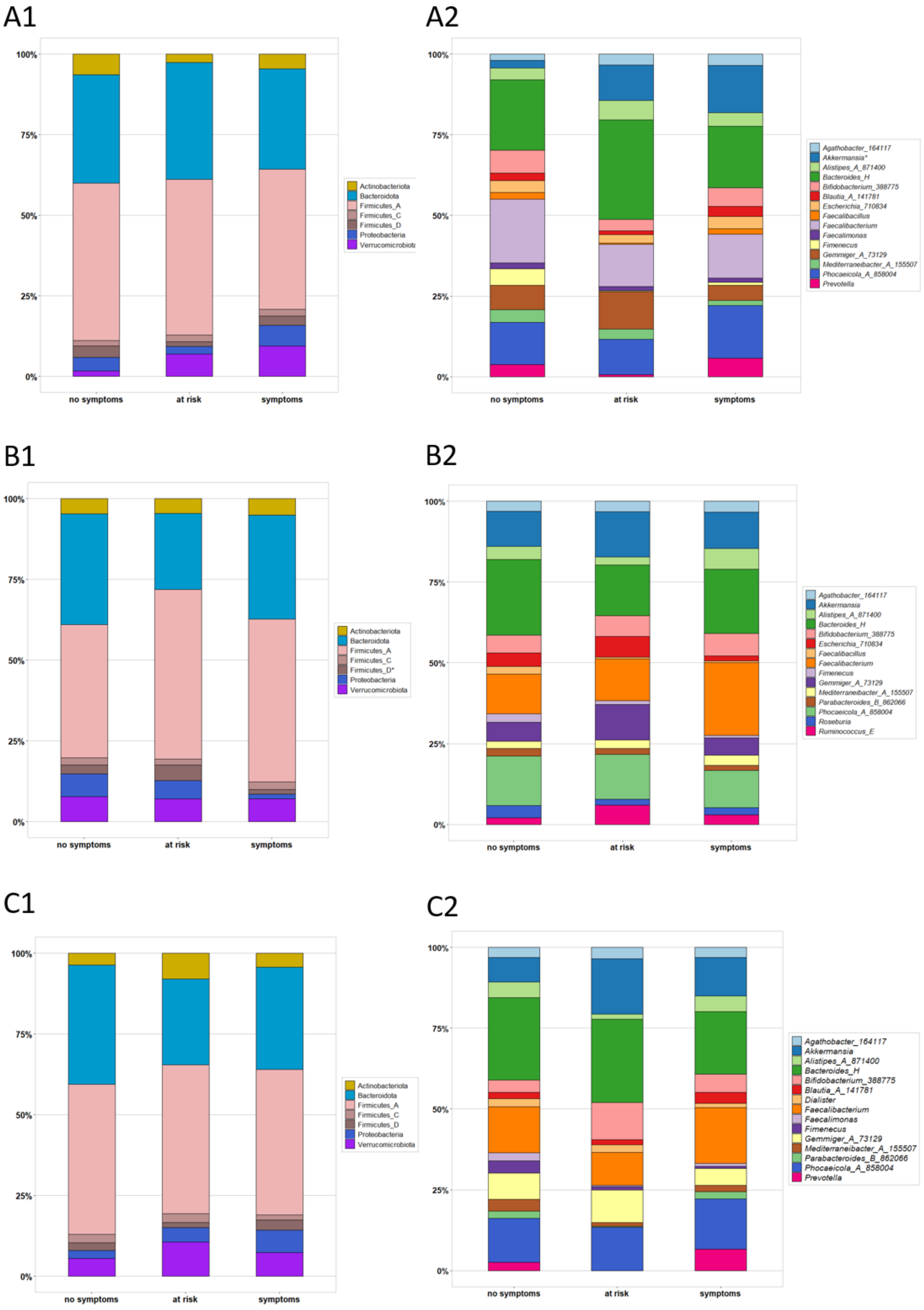

Supplementary Figure 5. Gut microbiota profiles of ASD patients stratified by CBCL INT (A) and CBCL_EXT (B) and CBCL_TOT (C). Abundance of phyla (A1, B1 and C1 panels) and top 15 genera (A2, B2 and C2 panels) were expressed as relative percentage for ASD patients stratified by CBCL_INT, CBCL_EXT and CBCL_TOT neuropsychological features.
Kruskal-Wallis test. *p-value < 0.05.
