# Supplementary material for: Stratification of Gut Microbiota Profiling Based on Autism Neuropsychological Assessments
Source: Microorganisms. 2024 Oct 9;12(10):2041. doi: 10.3390/microorganisms12102041 (PMC11510388; doi:10.3390/microorganisms12102041)
Supplement: Supplementary file 1 [file microorganisms-12-02041-s001.zip › Figure S6.pdf]

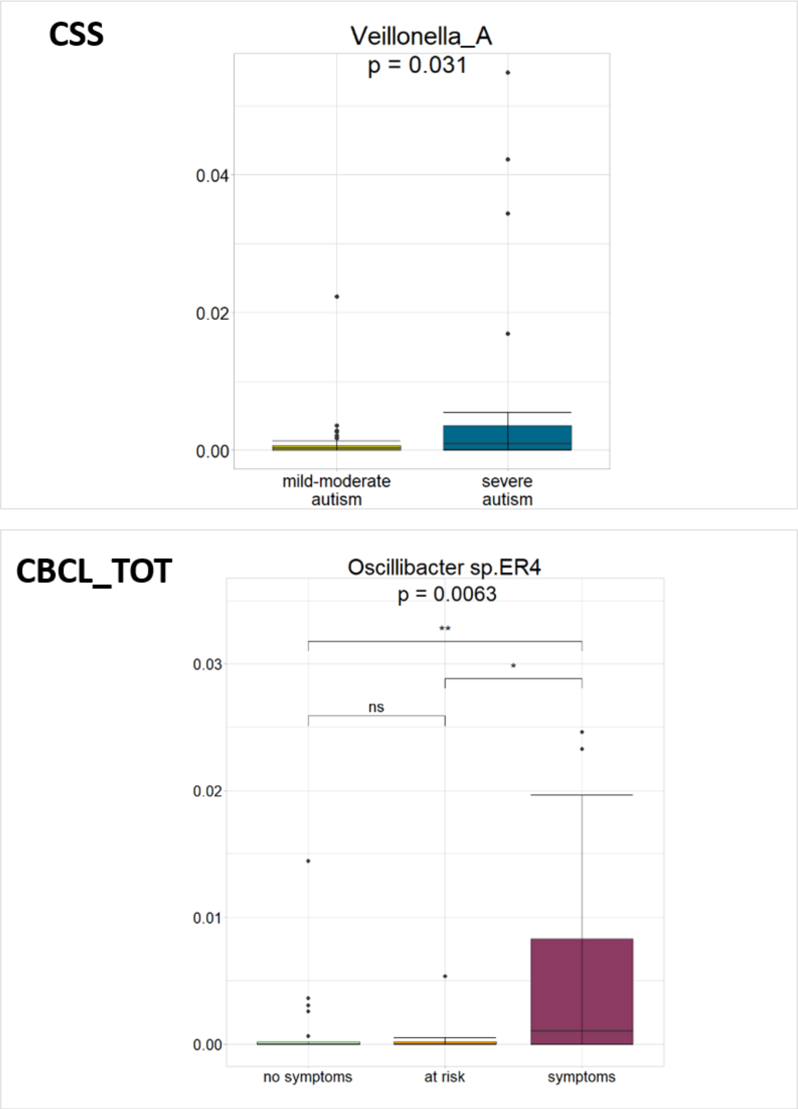

Supplementary Figure 6. Microbial biomarkers discriminating ASD patients grouped by neuropsychological features CSS and CBCL_TOT variables. Distribution of significant taxa at genus level between ASDs subgroups stratified by CSS and CBCL_TOT features (Mann-Whitney/Kruskal-Wallis test, p-value < 0.05).
