# Supplementary material for: Stratification of Gut Microbiota Profiling Based on Autism Neuropsychological Assessments
Source: Microorganisms. 2024 Oct 9;12(10):2041. doi: 10.3390/microorganisms12102041 (PMC11510388; doi:10.3390/microorganisms12102041)
Supplement: Supplementary file 1 [file microorganisms-12-02041-s001.zip › Figure S7.pdf]

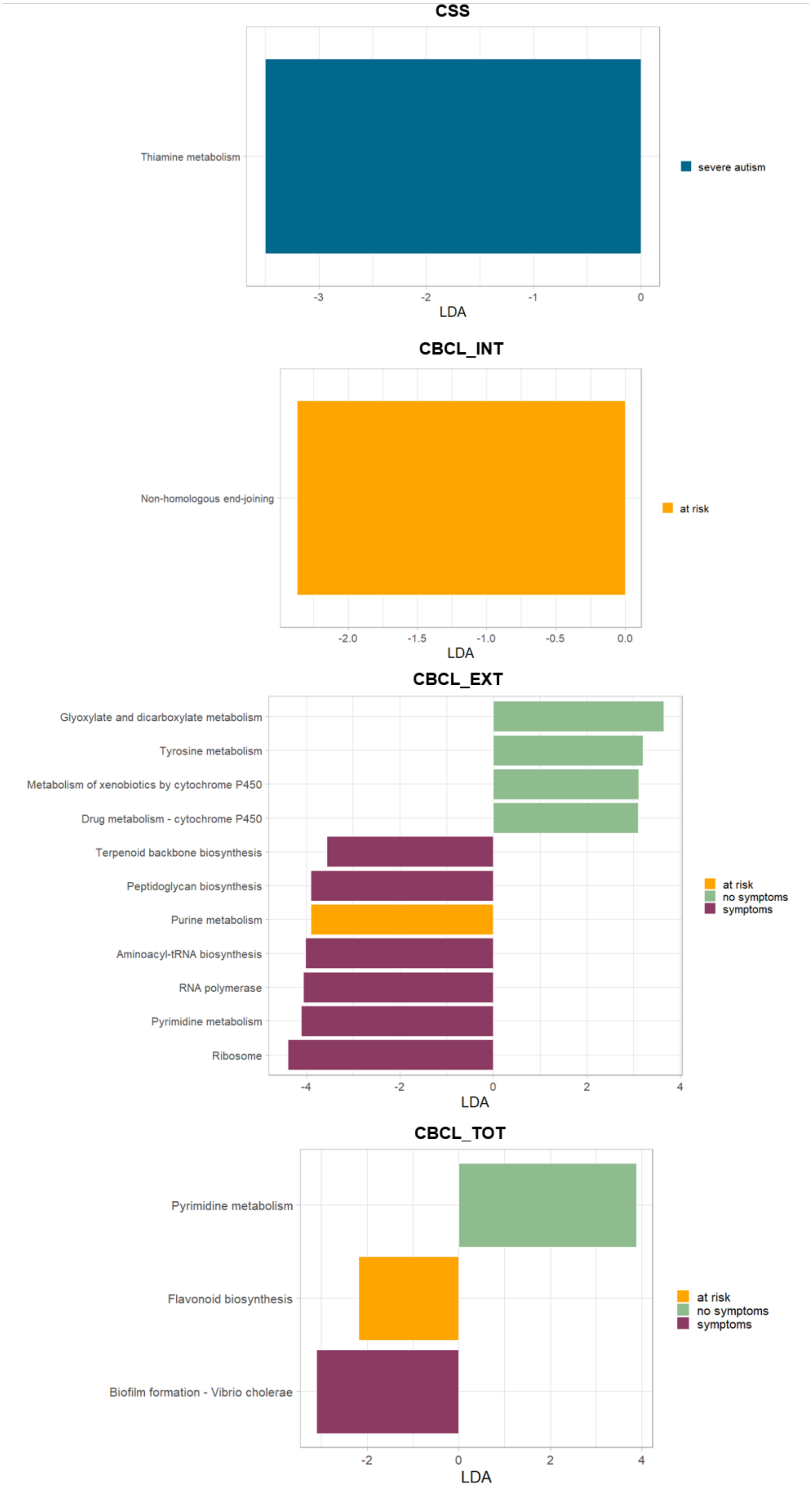

Supplementary Figure 7. Functional KEGG pathways predicted by Picrust2 algorithm discriminating ASD patients grouped by CSS, CBCL_INT, CBCL_EXT and CBCL_TOT. Statistically significant functional pathways (p-value < 0.05) were identified for ASDs patients stratified by CSS, CBCL_INT, CBCL_EXT, CBCL_TOT by limma voom method and LefSe analysis (LDA > 2).
