# Supplementary material for: Stratification of Gut Microbiota Profiling Based on Autism Neuropsychological Assessments
Source: Microorganisms. 2024 Oct 9;12(10):2041. doi: 10.3390/microorganisms12102041 (PMC11510388; doi:10.3390/microorganisms12102041)
Supplement: Supplementary file 1 [file microorganisms-12-02041-s001.zip › Figure S8.pdf]

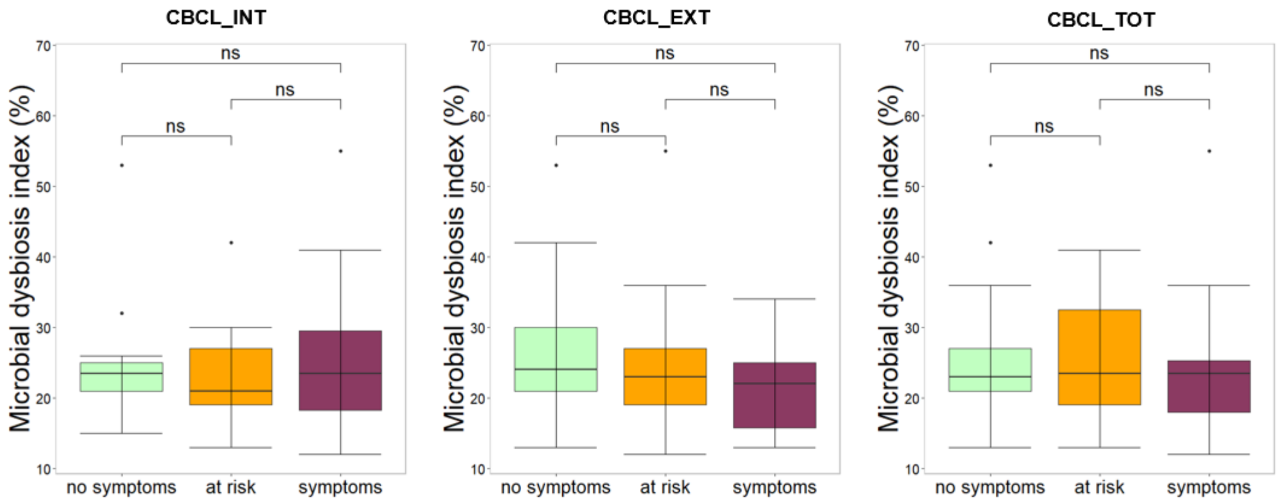

Supplementary Figure 8. MDI distribution in patients grouped by CBCL scores**.** Boxplot of MDI distribution (percentage) in patients stratified by CBCL_INT, CBCL_EXT, CBCL_TOT features. The pairwise-comparisons were obtained by a post hoc Mann–Whitney test (ns: $p > 0.05$).
